# Spermatic Cord Lipoma—A Review of the Literature

**DOI:** 10.3389/fsurg.2020.00039

**Published:** 2020-07-23

**Authors:** Ferdinand Köckerling, Christine Schug-Pass

**Affiliations:** Department of Surgery and Center for Minimally Invasive Surgery, Academic Teaching Hospital of Charité Medical School, Vivantes Hospital, Berlin, Germany

**Keywords:** inguinal hernia, lipoma, recurrence, pseudo-recurrence, spermatic cord lipoma

## Abstract

**Introduction:** A spermatic cord lipoma is found in 20–70% of all inguinal hernia repairs. The clinical picture of an inguinal hernia with bulging and pain but without an actual indirect hernia sac may become manifest in up to 8% of these cases. Missed spermatic cord lipoma can result in recurrence or pseudo-recurrence. This review presents the relevant literature on this topic.

**Materials and Methods:** A systematic search of the available literature was performed in February 2020 using Medline, PubMed, Google Scholar, Scopus, Embase, Springer Link, and the Cochrane Library, as well as a search of relevant journals and reference lists. Forty-two publications were identified as relevant for this topic.

**Results:** Spermatic cord lipoma seems to originate from preperitoneal fatty tissue within the internal spermatic fascia in topographical proximity to the arteries, veins, lymphatics, nerves, and deferent duct within the spermatic cord. Reliable diagnosis cannot be made clinically, but rather with ultrasound, CT, or MRI. In the absence of a real hernia sac, a spermatic cord lipoma is classified as a lateral inguinal hernia with a defect size <1.5 cm according to the European Hernia Society (EHS LI). Missed or inadequately treated spermatic cord lipoma results in recurrence or pseudo-recurrence. Since spermatic cord lipoma obtains its vascular supply from the preperitoneal space, it can be reduced or resected.

**Conclusion:** Spermatic cord lipoma is a common finding in inguinal hernia repairs and must be properly diagnosed and treated with care respecting the anatomy of the spermatic cord.

## Introduction

Lipomas are often found in the inguinal canal during surgical repair of inguinal hernias (1). In the literature, these are known as “lipomas of the cord,” “lipomas of the round ligament,” “spermatic cord lipomas,” and “inguinal cord lipomas” (1–3). Spermatic cord lipomas can either accompany a lateral inguinal hernia or occur without a synchronous hernia (1–3). They must be differentiated from the characteristic preperitoneal fatty tissue seen in a direct inguinal or femoral hernia (1).

A spermatic cord lipoma together with the hernia sac may increase the size of an indirect inguinal hernia and aggravate the symptoms. Furthermore, a spermatic cord lipoma may mimic the diagnosis and symptoms of inguinal hernia without the presence of an additional indirect hernia sac (1–4).

Missed or inadequately treated spermatic cord lipomas may cause unfavorable outcomes necessitating reoperation (1–4). Therefore, this paper now reviews and interprets the literature available on spermatic cord lipomas.

## Methods

**Figure 1:**
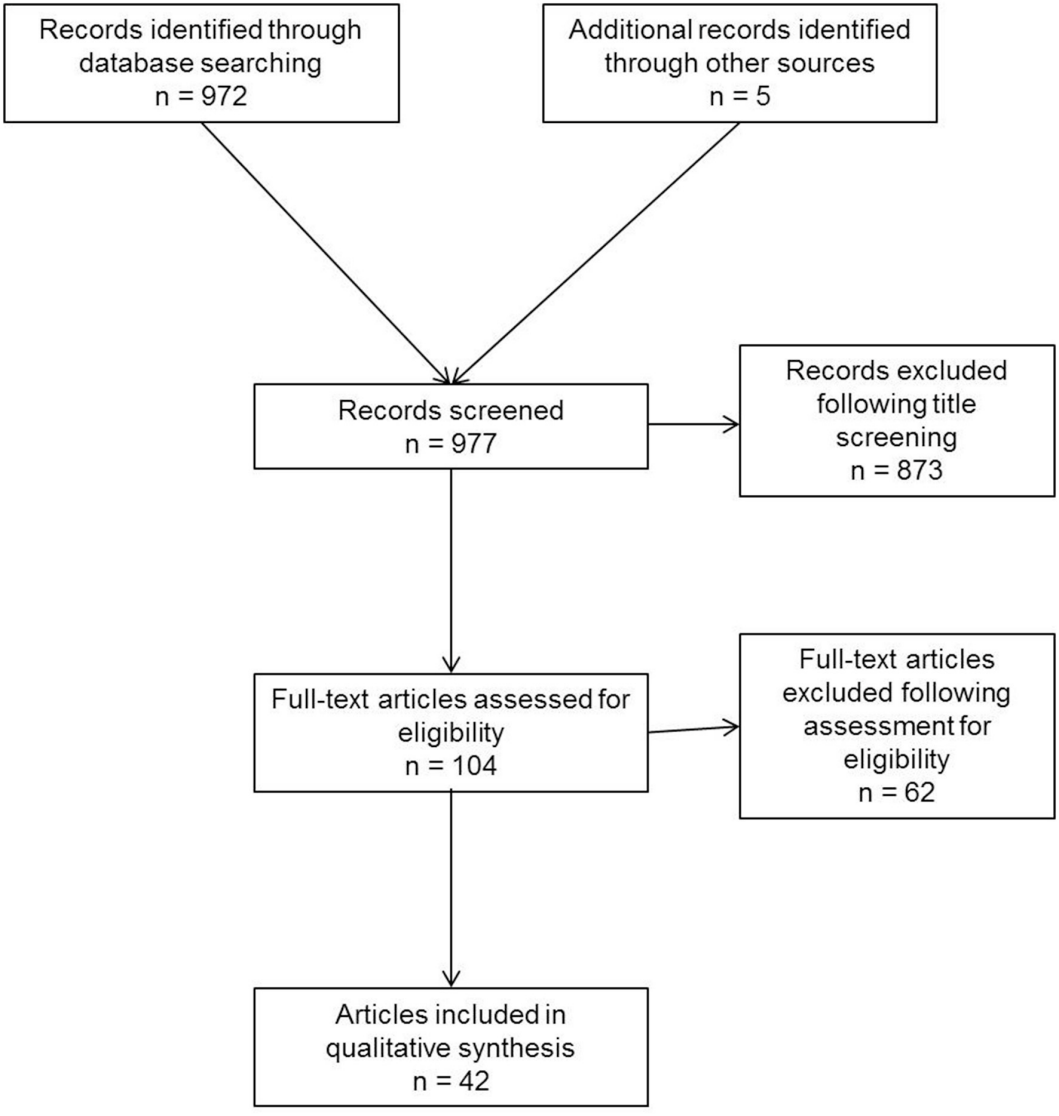
Flowchart of study inclusion.

A systematic search of the available literature was performed in February 2020 using Medline, PubMed, Google Scholar, Scopus, Embase, Springer Link, and the Cochrane Library, as well as a search of relevant journals and reference lists. The following search terms were used: “inguinal canal lipoma,” “preperitoneal fatty tissue,” “inguinal cord lipoma,” “cord lipoma,” “round ligament lipoma,” “spermatic cord lipoma,” “cord lipoma and recurrence,” and “cord lipoma and pseudo-recurrence.” The titles and abstracts of 977 publications were screened (Figure 1). For the present analysis, 42 publications were identified as relevant for this topic.

### Anatomical Background

**Figure 2:**
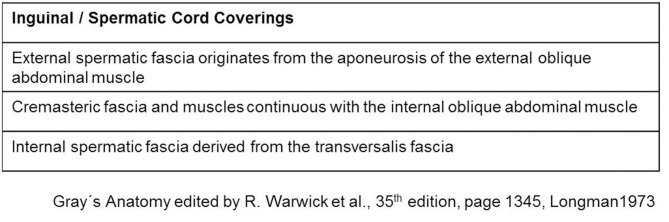
Coverings of the spermatic cord.

**Figure 3:**
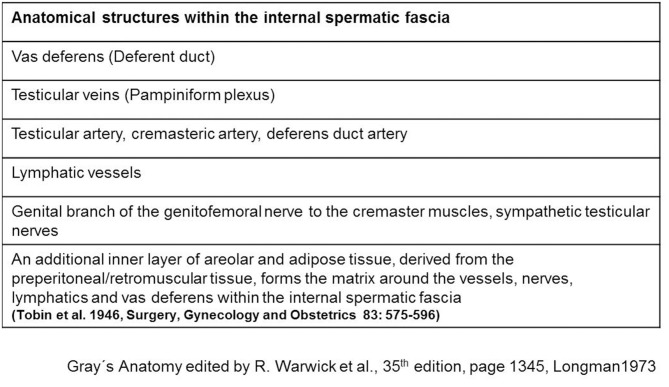
Anatomical structures within the internal spermatic fascia.

**Figure 4:**
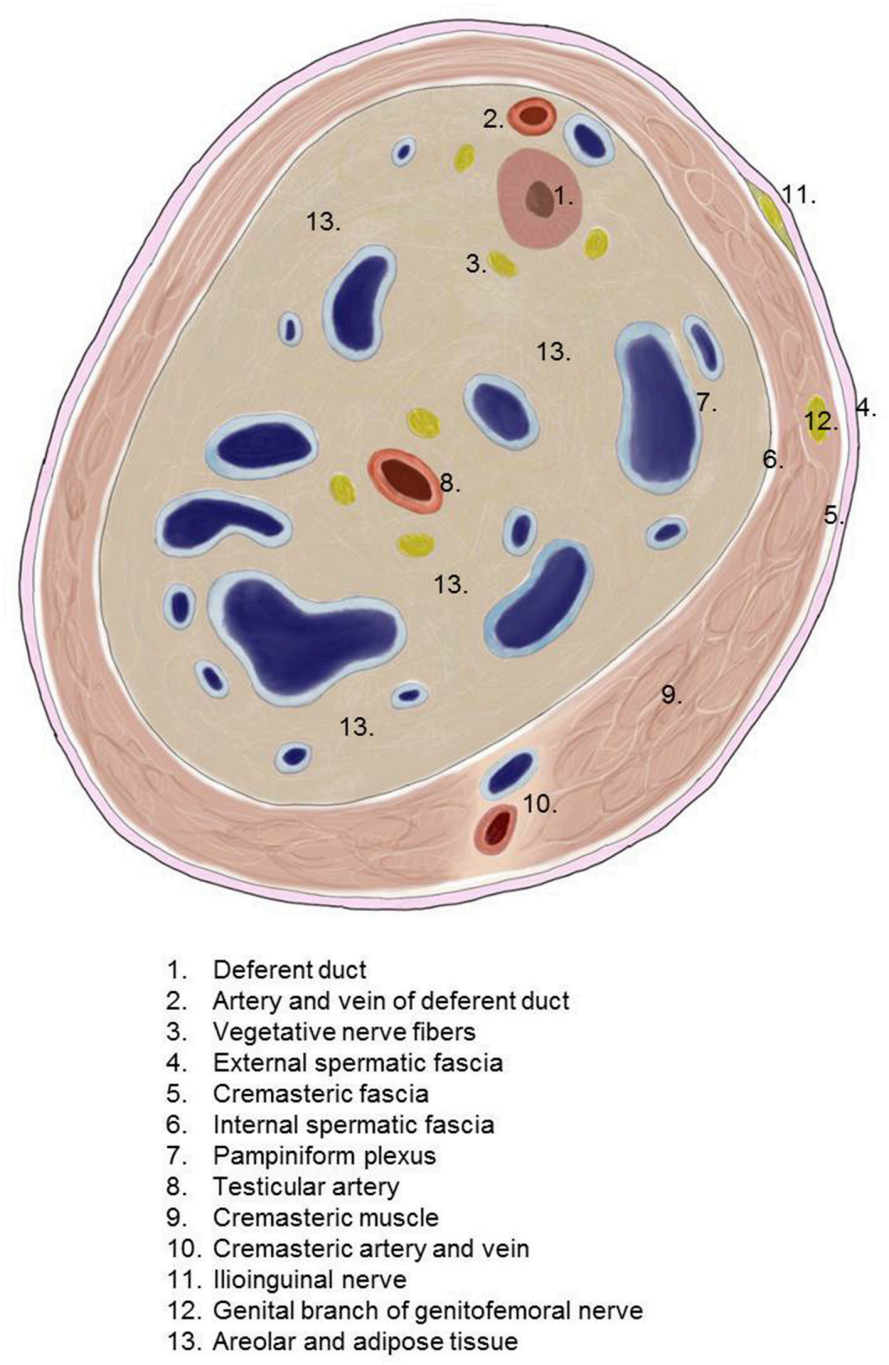
Transverse section of the spermatic cord (modified according to 7).

The spermatic cord traverses the inguinal canal and has three coverings derived from the layers of the abdominal wall (5) (Figure 2). The external spermatic fascia is a thin fibrous stratum originating from the aponeurosis of the external oblique abdominal muscle (5). The cremasteric fascia consists of a number of muscular fasciculi, united to one another by areolar tissue, and is continuous with the internal oblique abdominal muscle (5). The internal spermatic fascia is a thin layer derived from the transversalis fascia (5). The internal spermatic fascia encloses the arteries, veins, lymphatics, nerves, and the deferent duct that are united to one another by areolar and adipose tissue (Figure 3) (5, 6). According to Tobin et al. (6), an additional inner layer of areolar and adipose tissue, derived from the preperitoneal/retromuscular tissue, forms the matrix around the vessels, nerves, lymphatics, and vasa deferentia within the internal spermatic fascia (Figure 4) (7).

### Etiology of the Inguinal/Spermatic Cord Lipoma

In the cadaver study by Heller et al. (8), the inguinal/spermatic cord lipoma was “always continuous with the preperitoneal fat through the deep inguinal ring” (8). “The spermatic cord lipoma showed a characteristic pedunculated form with a bulbous distal end in the majority of cases” (8). “The typical appearance of the fat mass is that of a well-defined pedunculated mass lying in the long axis of the inguinal canal and arising from the preperitoneal fat” (8). The inguinal/spermatic cord lipoma is “located deep to the cremaster muscle and fascia” (8). Based on this characteristic anatomical appearance, the etiology of inguinal cord lipoma was described in the hypothesis by Heller et al. (8) as follows: “This anatomical configuration would be compatible with the mass arising simply as the result of gravitational force on semi-fluid preperitoneal fat, the characteristic pedunculated form being produced by the constraining effects of the internal oblique muscle and the cord or spermatic fasciae.” This is not a true “lipoma,” which is a benign tumor of adipocytes confined to the inguinal canal and shows an absence of connection with retroperitoneal fat (8). “True” lipoma can also develop in the retroperitoneum or intraabdominally and can become a malignant liposarcoma, but since the term spermatic cord lipoma has become established in the clinical setting, it continues to be used. The spermatic cord lipoma is really preperitoneal fatty tissue that gained access to the spermatic cord where it is united with the inner matrix of areolar and adipose tissue within the internal spermatic fascia (6, 8). These anatomical and etiological insights are particularly important when dissecting spermatic cord lipomas, because the fatty tissue is in close contact to the anatomical structures within the internal spermatic fascia of the cord.

### Prevalence of Spermatic Cord Lipomas in Post-mortem Groin Dissections

In a post-mortem dissection study of 36 body halves, a discrete mass of fat was seen within the inguinal canal in 75% of cases (8). In all cases, the fat mass in the spermatic cord was united to the preperitoneal fatty tissue via the inner inguinal ring (8).

### Clinical Prevalence of Spermatic Cord Lipoma

Fawcett et al. (9) found 46 inguinal cord lipomas when performing 140 inguinal hernia repairs in 129 patients (33%). Lilly and Arregui (1) reported 32 spermatic cord lipomas in 137 indirect inguinal hernias (23%). Nasr et al. (10) found 26 spermatic cord lipomas in 123 inguinal hernia repairs (21.1%). Carilli et al. (11) identified 100 (71.9%) spermatic cord lipomas in 128 patients with 139 indirect inguinal hernias who had undergone open surgery. Lau et al. (12) found 132 (26.5%) spermatic cord lipomas in 498 unilateral and 112 bilateral inguinal hernia repairs in totally extraperitoneal patch plasty technique (TEP). As such, the incidence of spermatic cord lipoma, with and without hernia sac, is likely to be seen in 20–70% of inguinal hernias. From clinical experience, the spermatic cord lipoma seems to develop often at the lateral corner of the deep ring, but this is not their “birthplace.”

### Clinical Prevalence of Spermatic Cord Lipoma Without an Indirect Hernia Sac

In a laparoscopic study with 2190 inguinal hernia repairs, Hollinsky and Sandberg (4) found normal groins with no sign of peritoneal defects or hernia sacs and lipomas of the cord or round ligament in 46 cases (2.1%). Fawcett and Rooney (9) reported only fatty protrusion in 3 of 112 (2.7%) inguinal hernia repairs. In the study by Nasr et al. (10), the incidence of pure inguinal canal lipomas without a hernia sac was 8.1%. Lau et al. (12) found a spermatic cord lipoma without a hernia sac only in 6 of 610 (1%) inguinal hernia repairs in TEP technique. Yener et al. (13) reported 22 spermatic cord lipomas without a hernia sac in 768 (2.9%) patients with indirect inguinal hernia.

The prevalence of spermatic cord lipoma without a visible hernia sac is thus 1–8%.

### Round Ligament Lipoma

Lilly and Arregui (1) found a lipoma of the round ligament in 9 of 25 inguinal hernia repairs in women (36%). Lau et al. (12) identified that only in 12.5% of female inguinal hernia repairs. Without evidence of an additional hernia sac, Yener et al. (13) reported only 1.4% round ligament lipomas in female inguinal hernia repairs.

### Differentiation From “True” Lipomas

Spermatic cord lipomas are the most common benign tumors in the inguinal canal (14–17). The term “true” lipoma may only be applied to those lipomas confined to the inguinal canal and not continuous with the preperitoneal or retroperitoneal fatty tissue. Primary tumors can develop from the tissues of the inguinal canal and spermatic cord (14–17).

### Classification of Spermatic Cord Lipoma Without a Hernia Sac

Even those spermatic cord lipomas without an additional indirect hernia sac can cause the typical inguinal hernia symptoms with bulging and pain (1, 10, 13). They should therefore be viewed and treated like “true” inguinal hernias (1, 10, 13).

The European Hernia Society (EHS) groin hernia classification therefore recommends that inguinal hernias without a hernia sac but with a lipoma in the inguinal canal should be classified as EHS L1 (lateral or indirect inguinal hernia) with a defect size <1.5 cm (18). That enables an exact EHS classification of these findings seen in 1–8% of all inguinal hernias.

### Clinical Diagnosis

In general, preoperative clinical examination does not facilitate identification of the presence of a spermatic cord lipoma. Even without a hernia sac, spermatic cord lipomas may give rise to the clinical manifestations and symptoms seen in an inguinal hernia with a hernia sac (1, 10, 13). This should always be clinically suspected when patients report inguinal pain in the absence of inguinal protrusion (1). If there is a discrepancy between the preoperative inguinal hernia size identified on clinical examination and that seen intraoperatively, an intensive search must be undertaken for a lipoma deep in the inguinal canal.

### Ultrasound

A lipoma can be seen as a hyperechoic mass on sonography (19–24). If there is no inguinal protrusion on clinical examination, ultrasound has a 75% accuracy in identifying groin pathologies (21). Differential diagnosis of fat-containing lesions seen on ultrasound must consider the echogenicity, echostructure, and vascularity (22). The absence of connection with abdominal fat and the usual absence of vascularization in color Doppler are important features of a true lipoma (22). Missed or unresected spermatic cord lipomas can also be diagnosed as recurrences or pseudo-recurrences on postoperative ultrasound (24, 25). Preoperative ultrasound examination is useful for diagnosis of spermatic cord lipoma to avoid reoperations (3).

### Computed Tomography

Spermatic cord lipomas can also be reliably diagnosed with computed tomography (26, 27). However, whether a hernia sac is present or not cannot be reliably differentiated on CT (26). In a series of 49 patients with inguinal hernia diagnosis who had undergone surgery, three had only a spermatic cord lipoma without a hernia sac (26).

### Magnetic Resonance Imaging

MRI is recommended primarily in the literature to differentiate large lipomas and tumors in the inguinal canal (28–32). MRI is also useful in differentiating soft tissue abnormalities in settings of an unclear hernia diagnosis (17). No specific study findings are available on the role of MRI in diagnosis of spermatic cord lipoma.

### Different Clinical Scenarios

The presence of lipomas in the inguinal canal or in the spermatic cord may result in very different clinical scenarios.

- Giant groin lipoma mimicking an inguinal hernia.

Lipomas in the groin can grow to such a size as to be mistaken for inguinal hernia (28–32). If groin tumor rather than inguinal hernia is clinically suspected, thorough diagnostic workup is needed with CT or MRI (28–32). Other pathologies of the groin should be excluded in differential diagnosis: benign and malignant tumors, congenital abnormalities, vascular conditions, and infections or inflammatory processes (14, 15). In any suspicion of malignancy, an incisional biopsy or a true-cut biopsy is required prior to surgical excision. If diagnosis reveals a giant (huge) pure lipoma, it can generally be easily resected using an open route (28–32).

- Spermatic cord lipoma without a hernia sac

Only a spermatic cord lipoma without a real hernia sac was identified in open or laparo-endoscopic repair in up to 8% of clinically diagnosed inguinal hernias (4, 9, 10, 12). That problem arises if spermatic cord lipoma causes bulging even in the absence of a hernia sac (33–37). If a spermatic cord lipoma is not completely resected or dissected or even missed due to incomplete exploration of the inguinal canal, it may result in recurrence or pseudo-recurrence (33–40). Therefore, based on the European Hernia Society, spermatic cord lipoma without lateral hernia sac is classified as lateral or indirect inguinal hernia (EHS L1) (18).

A Consensus Development Conference of the European Association of Endoscopic Surgery (EAES) has recommended an active search for spermatic cord lipoma in all laparo-endoscopic inguinal hernia repairs (41). It also recommends that the spermatic cord lipoma should be reduced (41).

Likewise, the guidelines for laparoscopic (TAPP) and endoscopic (TEP) treatment of inguinal hernia of the International Endohernia Society (IEHS) state that spermatic cord lipoma may imitate primary hernia, hernia recurrence, or become symptomatic in the later course (42, 43). Therefore, the IEHS guidelines recommend removal of the spermatic cord lipoma (42, 43).

- Discrepancy between a preoperative extent of bulging and the intraoperative size of the hernia sac

If there is a noticeable difference between the preoperative extent of the inguinal hernia bulging diagnosed by the surgeon and the intraoperative size of the lateral hernia sac, the presence of an additional spermatic cord lipoma should be suspected. In such cases, a meticulous search must be performed in both open and laparo-endoscopic inguinal hernia surgery deep in the inguinal canal for a spermatic cord lipoma. That is also generally identified as such. In such settings too, spermatic cord lipoma should be reduced or resected to avoid recurrence or pseudo-recurrence.

### Operative Technique

**Figure 5:**
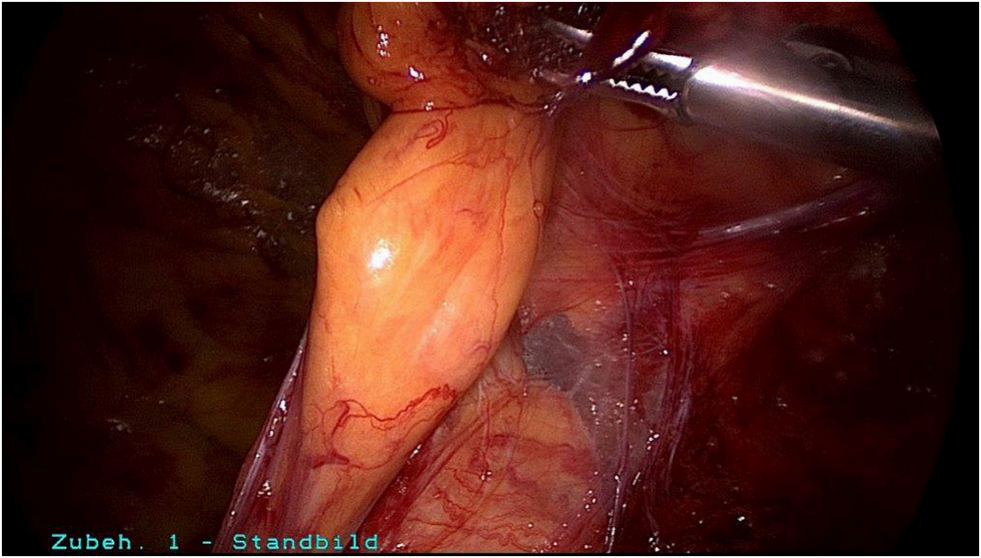
Spermatic cord lipoma in a patient with bulging and groin pain without indirect hernia sac.

**Figure 6:**
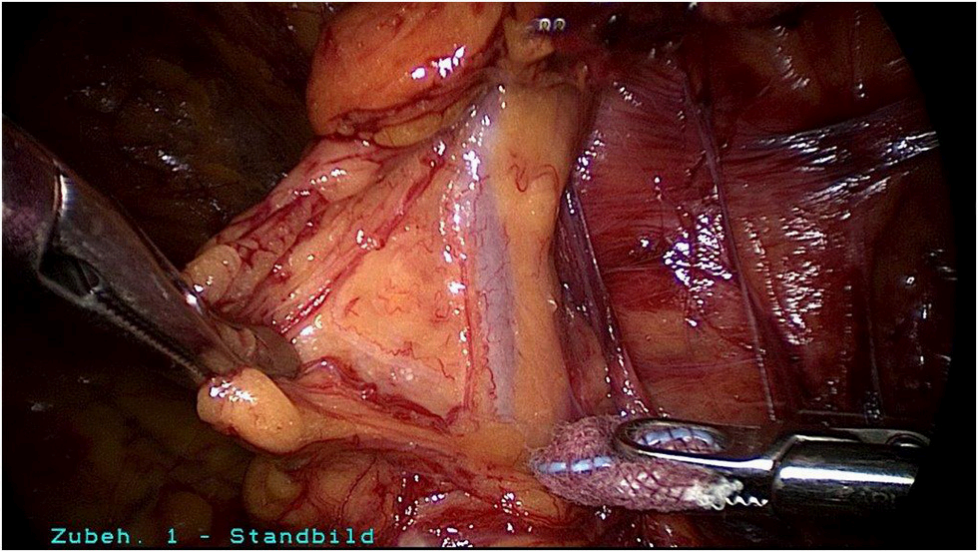
Fatty tissue around the vessels in the spermatic cord.

**Figure 7:**
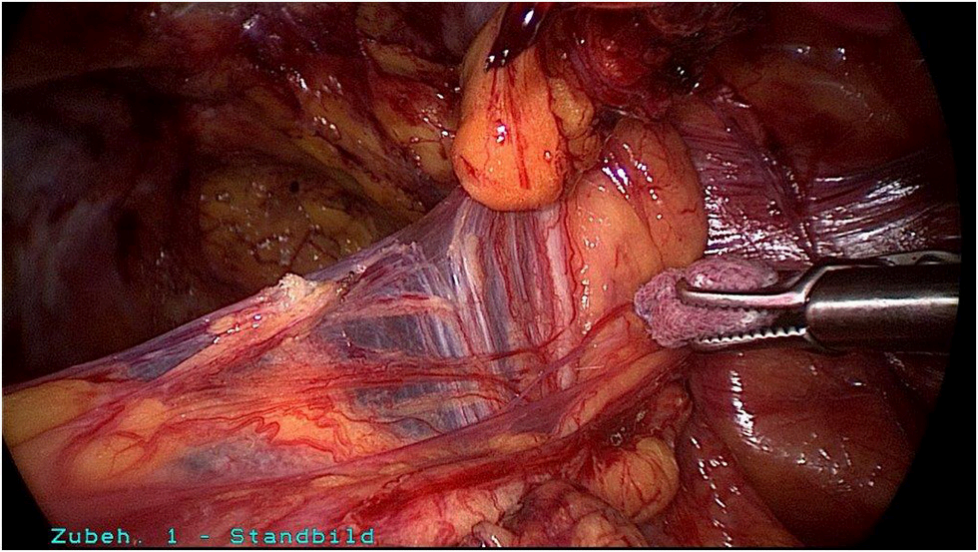
Blunt dissection between deferent duct and the spermatic cord lipoma.

**Figure 8:**
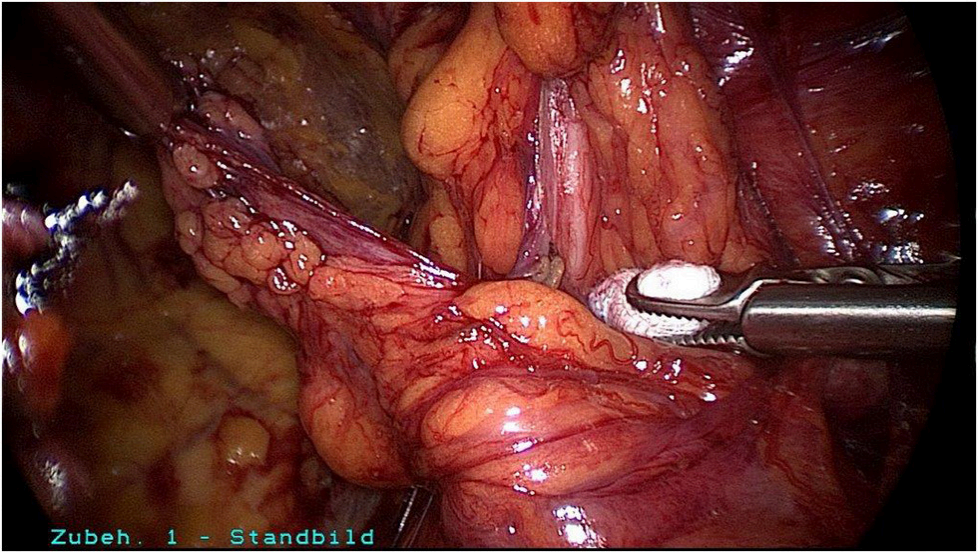
Spermatic cord lipoma after reduction in the preperitoneal space.

Surgical reduction or resection is technically demanding. As explained above, the spermatic cord lipoma originates from preperitoneal fatty tissue within the spermatic cord enclosed by the internal spermatic fascia with the arteries, veins, lymphatics, nerves, and deferent duct. These anatomical structures are united to one another by a matrix of areolar and fatty tissue. Accordingly, there is close topographical proximity between the spermatic cord lipoma and the anatomical structures enclosed by the internal spermatic fascia. That makes lipoma dissection from the spermatic cord structures challenging. The lipoma must be bluntly dissected from the deferent duct, arteries, veins, lymphatics, and nerves while preserving the surrounding fatty tissue. The dissection boundary to the deferent duct can be relatively well visualized with a swab using blunt dissection (Figure 5). It is more difficult to identify the boundary between the fatty tissue enclosing the spermatic cord structures and the lipoma. Extreme caution is needed here to protect the spermatic cord structures (Figures 6–8).

The presence of a spermatic cord lipoma and its treatment should be mentioned in the operation chart.

Since the spermatic cord lipoma has its origin in, and thus obtains its vascular supply from, the preperitoneal space, it can be either reduced, i.e., dissected from the spermatic cord and left, or resected. The specific approach taken will depend on the anatomical structures and the feasibility of mesh placement.

Lau et al. (12) reported on 119 inguinal/spermatic cord lipomas with reduction “to pelvic peritoneal reflection line after division of the feeding vessels from surrounding structures” and on 13 resections. The repairs were performed in totally extra-peritoneal patch plasty technique. The outcome did not differ from that of TEP repair in patients with inguinal hernia without evidence of a spermatic cord lipoma (12).

## Discussion

According to a hypothesis by Heller et al. (8) spermatic cord lipomas are fat masses originating from the preperitoneal fatty tissue within the internal spermatic fascia of the spermatic cord. Due to its location within the internal spermatic fascia, the spermatic cord lipoma has a close topographical relationship to the deferent duct, pampiniform plexus, testicular artery, cremasteric artery, deferential artery, lymphatics, genital branch of the genitofemoral nerve, and ilioinguinal nerve.

A spermatic cord lipoma can give rise to a clinical picture with bulging and pain mimicking that of inguinal hernia, even in the absence of a hernia sac. If an indirect hernia sac is also present, the spermatic cord lipoma will increase the size of the bulging clinically diagnosed.

In 20–70% of all inguinal hernia repairs, a spermatic cord lipoma is detected either together with an indirect hernia sac or without a hernia sac. The highest prevalences reported were 75% in a post-mortem dissection study (8) and 71.9% in a clinical study of open inguinal hernia repair (11). In clinical studies of laparo-endoscopic inguinal hernia surgery, the prevalence was only 20–30% (1, 10, 12). It might be possible that the detection rate of spermatic cord lipoma is lower in laparo-endoscopic inguinal hernia repair compared to open surgery and the true prevalence of spermatic cord lipoma might be higher. In addition, even a higher prevalence may not be clinically significant since most lipomas are very small.

The prevalence of spermatic cord lipoma without a visible hernia sac is 1–8%.

The prevalence of round ligament lipoma at 1.4–36% (1, 12, 13) is markedly lower. The round ligament is a band of connective tissue that supports the uterus and is accompanied by feeding vessels. Unlike the spermatic cord, it does not have an inner matrix of adipose tissue derived from the preperitoneal fatty tissue. That is thought to explain the difference in prevalence.

Spermatic cord lipomas are categorized as per the EHS classification system, even in the absence of a hernia sac, as indirect groin hernia with a defect size of <1.5 cm (EHS L1) (18). It remains unclear why a large lipoma with an inner hernia opening of more than 1.5 cm should not be categorized with respect to the width of the opening. This should be at least discussed in the future.

Spermatic cord lipomas are the most common benign tumors seen in the inguinal canal (14–17). Spermatic cord lipoma cannot be reliably diagnosed through clinical examination. Only ultrasound, CT, and MRI are able to assure reliable preoperative diagnosis of spermatic cord lipoma (19–24). The criteria used to distinguish between a spermatic cord lipoma and a “true” lipoma are the absence of a connection between the “true” lipoma and the preperitoneal fatty tissue as well as the usual absence of vascularization in color Doppler (22). Liposarcomas are also typically continuous with the preperitoneal tissue (19–24). Therefore, if a malignant tumor is suspected, an incisional biopsy or a true-cut biopsy should be performed as a first step.

According to the international HerniaSurge guidelines, a typical, symptomatic inguinal hernia protrusion seen on clinical examination constitutes an indication for surgery (44). Further diagnostic workup based on ultrasound, CT, or MRI is generally not needed (44). However, under consideration of the high prevalence of the spermatic cord lipoma, the indication to perform ultrasound examination in every patient with an obvious or suspected inguinal hernia should be discussed.

Hence, surgery is also indicated for patients without a typical inguinal protrusion but with inguinal pain and evidence of a spermatic cord lipoma on ultrasound, CT, or MRI (18, 44).

Spermatic cord lipoma without inguinal complaints detected as an incidental finding should be kept under observation.

Any discrepancy between the intraoperative indirect hernia sac identified and the preoperative clinical bulging is suggestive of an additional spermatic cord lipoma. This should always be ruled out because of the high prevalence of spermatic cord lipoma.

Missed spermatic cord lipoma results in recurrences or pseudo-recurrences. However, there are no data available indicating that even dissection of very small lipomas is safe due to the close connection to the vascular structures in the cord. Overtreatment should also be avoided.

In view of the close topographical relationship between the spermatic cord lipoma and the arteries, veins, lymphatics, nerves, and deferens duct enclosed by the internal spermatic fascia, dissection in the correct layer is difficult. Blunt dissection is recommended, while sparing the fatty tissue surrounding the spermatic cord structures. Since the fatty tissue of the spermatic cord lipoma has its origin in, and hence obtains its vascular supply from, the preperitoneal space, it can be either reduced or resected. Resection is only needed if post-reduction mesh placement is impeded. There are no reports in the literature that reduction of spermatic cord lipomas carries any risk of secondary infection by devascularized fatty tissue, thus supporting the hypothesis that spermatic cord lipomas obtain their main vascular supply from the preperitoneal space.

However, in view of the moderate quality of the available literature, careful consideration should be given to the recommendations.

## Author Contributions

FK and CS-P: literature research, literature review, manuscript writing, figure creation, final manuscript correction. All authors contributed to the article and approved the submitted version.

## Conflict of Interest

The authors declare that the research was conducted in the absence of any commercial or financial relationships that could be construed as a potential conflict of interest.
